# Correlation between serum leptin level and thyroid hormones in children with major beta-thalassemia 

**Published:** 2013-10-22

**Authors:** I Shahramian, NM Noori, AA Ramezani, E Sharafi, E Akhlaghi

**Affiliations:** 1 ^Fellow of Pediatric Gastroentrology, Shiraz University of Medical Sciences, Shiraz, Iran.^; 2 ^Professor of pediatric cardiology, Children and Adolescents Health Research Center, Zahedan Medical University, Iran ^; 3 ^School of health, Zabol Medical University, Zabol, Iran. ^; 4 ^Resident of Ophtalmology, Zahedan Medical University, Zahedan, Iran ^; 5 ^School of medicine, Zabol Medical University, Zabol, Iran.^

**Keywords:** Thalassemia, leptin, thyroid hormone, thyroid stimulating hormone

## Abstract

**Background:**

Beta-thalassemia is the most common hematology disease in human and leptin is one of the hormone that produce by adiposities cells. The purpose of this study was to investigate the relationship between serum leptin level and thyroid hormones in children with major beta-thalassemia.

**Materials and Methods:**

This descriptive-cross sectional study was performed on 90 children aged 6-16 years old with beta-thalassemia. Body Mass Index (BMI ) were meuseurd in all patients and then, after collecting the samples, leptin and thyroid hormones levels of the serum were measured in the patients with thalassemia via ELISA method. Then, all data was analyzed by Pearson correlation test, and x2 statistical tests and P < 0.05 was considered as a significant difference.

**Results:**

The mean of body mass index and serum leptin level in the patients group was 16.58±2.43 and 1.521 ±2. 49, respectively.

The mean serum levels of thyroxin (T4), triiodothyronine (T3), and thyroid- stimulating hormone (TSH) in patient's groups were7.94 ±3.56, 1.28 ± 0.46, and 2.85 ±3. 44, respectively. There was significant correlation between serum leptin levels and T4 in patients with major thalassemia; also there was no significant correlation between serum leptin level and T3and TSH. There was a significant correlation was between the leptin serum level and BMI in patients (P value=0.008).

**Conclusion:**

The results of this study demonstrated that in patients with major thalassemia, there was significant correlation between serum leptin level and thyroxin hormone. Leptin level has more relationship with thyroxin than thyroid- stimulating hormone.

## Introduction

Thalassemia is the most common heterogeneous disease of human being, and every year, 100,000 neonates are born with hemoglobinopathies around the world. It is a disease of high prevalence in Mediterranean, Indian, North Chinese, and Pacific populations. Recently, the quantity and quality of the life of these patients have been significantly improved by regular transfusion and iron chelating therapy (1, 2). In another study, shahramian and et al showed that serum levels of leptin in major beta thalassemia reduces regardless of age and body mass (3).

Leptin is a 146 -amino acid polypeptide which is produced by adipocytes (4 , 5). The receptor of this hormone, located in different parts of the body, not only regulates lipid and energy homeostasis, but it also affects neuroendocrine and immune function (6-8). The hormone is regulated by a problematical complex consisted of several mediators, including insulin, Glucocorticoids and thyroid hormones (6). 

Fat cells in beta-thalassemia children are not able to synthesize adequate amounts of leptin(9). Adipose tissue dysfunction is one of the reason for hormonal abnormalities in major beta thalassemia patients. Leptin is mainly produced in the hypothalamus and the leptin deficiency causes premature consistent performance in the pituitary- hypothalamus (10). Its greatest influence is on the hypothalamus caused by JAK STAT signal. This hormone probably affects the thyroid axis in hypothalamus by binding to its Long receptor in the hypothalamus, and activates Jak state. Also, its impact on the para-ventricular cores is effective for TRH gene regulation (11, 12).

Due to mutations and delay in the maturation of red blood cells, children with major beta-thalassemia may have face various effects and metabolic, endocrine, or neurological abnormality which occurs in these patients due to repeated transfusion (13-15). Frequent transfusion is mostly accompanied with iron overloading and its accumulation in various tissues, including thyroid (16). Iron accumulation causes reactive oxygen in erythrocytes, oxidative stress, peroxides, and releases radicals. A number of the effects of oxidative stress are endocrine, cardiovascular, and vascular complications (17). The endocrine effects and in fact, thyroid dysfunction and hypothyroidism lead to decreased metabolism, fatigue, increase in blood cholesterol levels, joint pain, heart problems, depression and attention disorders as well as periods of forgetfulness (12).

 Thyroid presentation takes place by T3 and T4 hormones whose secretion is incited or suppressed by TSH. The regulation and synthesis of TSH activity are regulated by TRH secreted from hypothalamic Para-ventricular nuclei (18-20). In major beta-thalassemia patients, due to disorders in the pituitary hypothalamic axis, we may observe endocrine disorders such as hypothyroidism.

The purpose of this study was to investigate the relationship between serum leptin level and thyroid hormones in children with major beta-thalassemia.

## Materials and Methods

In this descriptive-cross sectional study, 90 children with an age range between 6 to 16 years old, suffering from thalassemia, diagnosed by hemoglobin electrophoresis. These children selected by Haphazard sampling and among those referred to Zabol Amir-al-Momenin Hospital for receiving Packed Red Blood Cell. Among all studied children, 46were male and 44 were female. After history taking, physical examination, chest radiography, and electrocardiography (ECG), patients with mitral valve dysfunction, hypertension, structural diseases of the heart, heart failure, metabolic diseases, iron deficiency anemia, endocrine or renal disorders, hemoglobin before transfusion less than 9 g/dl, history of irregular transfusion before two years of age, and duration of chelating therapy less than 5 years were excluded from the study. From all of the children, 5cc blood was drawn at 8:00 am. Samples were centrifuged at 3000 g for 10 minutes at 5 ° C. Separated serum was kept at -20 fridges till measurement amount of thyroid hormones and leptin. Finally, under the cold chain, it was transferred to the Biochemistry Lab of Zabol University of Medical Sciences. Then, 250 micron of serum samples was isolated in order to analyze thyroid hormones by Diaplus Inc with ELISA method(US), and the other serum samples used for evaluation of leptin level by Diagnostic Biochem with ELISA method (Canada). 

Height(cm) and weight(Kg) were measured in all children. In this study, the children over 2years old were weighed using RASA Mark made in IR Iran by an error factor of 100, and the height of children above 2 years old was measured in the standing position with a scale ruler. Then BMI was calculated with (W Kg / Hm²) (weight in kilograms divided by height to the power of 2) formula. 

**Statistical analysis**

Data was collected using the SPSS software version 20, and descriptive- analytical statistics were Performed by statistical Pearson correlation analysis, and P <0.05 was considered as the significance level. 

## Results

In this study, 90 cases of children admitted to the pediatric department of Amir-al- Momenin hospital, Zabol (major beta thalassemia receiving a packed red blood cell). The mean BMI of the study population was 16.58+2.43. Mean leptin serum level in the study group was 1.521 +2. 49 Pg/ml. In these patients, the mean of T4, T3, and TSH serum levels were 7.94 +3.56 ng/dl, 1.28 + 0.46ng/dl, and 2.85 +3. 44 µIU/m l, respectively.

In this study, a significant difference was found between the serum leptin level and thyroxin (T4) level in children with major beta thalassemia (P value=0.05).

 In addition, a significant correlation was between the leptin serum level and body mass index (BMI) in patients with major beta thalassemia (P value=0.008). Nevertheless, no significant relationship was found between leptin serum level, triiodothyronine (T3), and thyroid-stimulating hormone (TSH) in patients with major beta thalassemia respectively(P value=0.708)and(P value= 0.575).

**Table I:** Correlation between leptin serum level and BMI, TSH, T3, and T4 in patients with major beta thalassemia

Parameters	T4	T3	TSH	BMI
	7.94 +3. 56 ng/dl	1.28 + 0.46 ng/dl	2.85 +3. 44 mIU/ml	16.58+2.43 kg/m^2^
Leptin	R=0.328 P=0.05	R=-0.066 P=0.708	R=-0.098 P=0.575	R=0.442 P=0.008
1.521 +2. 49 Pg/ml				

## Discussion

This study was designed to investigate the relationship between leptin serum level and thyroid hormones in children with major beta-thalassemia. Leptin serum level is associated with serum T4 levels in a manner that with increasing leptin serum level also increased thyroxin level in serum. But there was not significant correlation between leptin, T3 and TSH in serum. Also there was a significant correlation between leptin serum level and BMI in patients with major beta thalassemia.

Ina study performed by Dayar et al, in patients with major beta thalassemia showed thatthere was not correlation between serum leptin level and serum thyroid stimulating hormone. Also in this research, they revealed that there was relationship between body mass index and serum leptin level in this patients. This part of study was consistent with our study (20). In another study by Corbet et al on the lack of thyroid hormone in leptin serum concentration, showed that the role of thyroid hormones in controlling leptin synthesis and secretion appears to be of little (21). In a study done by Hsieh CJ et al on evaluation of leptin serum concentrations in patients with abnormal thyroid function, the role of thyroid hormones in regulation of leptin metabolism was introduced. Therefore, it seems that leptin serum level increased in patients with hypothyroidism (23). In another study by Bates et al, the role of leptin receptor signals on the neuroendocrine- function relationship between leptin and thyroid hormones was proposed (11). In fact, leptin plays an important role in transforming the milieu of T4 to T3. In addition, it has a regulatory effect on TSH (19)due to mutations which fat cells are not able to synthesize adequate amounts of leptin in thalassemia patients, therefore amounts of leptin serum level decreased in these patients. As a result, the peripheral conversion of T4 to T3 decreases, and it leads to increase in serum T4 rather than T3 (10). Based on the previous article and the direct correlation of T4 with leptin, it seems that leptin is more sensitive to TSH thanT4 and it is affected by T4 earlier than TSH, whenever it reaches an acceptable level, it can affect TSH axis, whereas leptin is low in these patients (9). Therefore, leptin increases along with an increase in T4 but due to the lack of adipose tissue, leptin level does not increase enough to affect TSH, hence, no relationship was found in these patients. In this study, there was not significant correlation between leptin serum level and thyroid-stimulating hormone in patients with major beta thalassemia. In a study by Zimmermann et al on circulating leptin and thyroid dysfunction, leptin was inversely associated with TSH (19). In another study by Feifan Guo and his colleagues "showed that the thyrotropin releasing hormone promoter is targeted by leptin in vivo and that leptin can probably regulate the thyroid axis directly without need for response from the arcuate nucleus" (12). In fact, the hypothalamic-pituitary axis was regulated under the impact of STAT3 signal. This signal is made by leptin, prolactin, IL6 and TNF and the production of these intermediates are determined by the genetic factors (24). As the study groups were patients with major thalassemia and it plays a role in the pathophysiology of thalassemia inflammation (14), it leads to an increase in TNF and IL6. On the other hand, in thalassemia patients also observed aspect a reduction in leptin partly due to the genetic defect (9) This possibility is suggested that TNF and IL6 affect leptin much more than pituitary- hypothalamus axis. In addition, leptin effect is mostly related to para-ventricular nuclei associated with TRH that has not assessable in this study. In the present study, according to the age, a significant relationship was found between body mass index (BMI) and leptin serum level. Choobineh H et al in a study on the evaluation of leptin levels in thalasemic patients noticed that leptin was not related to BMI, but it has a significant relationship with age(9). In another study by Suzuki et al performed on the relationship between obesity and serum markers of oxidative stress and inflammation in Japan, a relationship between leptin serum level and BMI was proposed in which an increased fat mass has led to an increase in leptin production(22)

## Conclusions

In patients with major beta thalassemia, there was a significant correlation between leptin serum level and thyroxin hormone. Leptin has more related to changes in thyroxin than thyroid- stimulating hormone, but additional more researches are recommended to prove such association.

## Conflict of interest

The authors certify that they have NO affiliations with or involvement in any organization or entity with any financial or non-financial interest in the subject matter or materials discussed in this manuscript.
